# A deep Tasman outflow of Pacific waters during the last glacial period

**DOI:** 10.1038/s41467-022-31116-7

**Published:** 2022-06-30

**Authors:** Torben Struve, David J. Wilson, Sophia K. V. Hines, Jess F. Adkins, Tina van de Flierdt

**Affiliations:** 1 Department of Earth Science and Engineering, Imperial College London, SW7 2AZ London, UK; 2 The Grantham Institute for Climate Change and the Environment, Imperial College London, SW7 2AZ London, UK; 3 Institute for Chemistry and Biology of the Marine Environment (ICBM), University of Oldenburg, 26129 Oldenburg, Germany; 4 Institute of Earth and Planetary Sciences, University College London and Birkbeck, University of London, WC1E 6BT London, UK; 5 Department of Geological and Planetary Sciences, California Institute of Technology, Pasadena, CA USA; 6 Lamont-Doherty Earth Observatory, Columbia University, Palisades, NY USA; 7 Department of Marine Chemistry and Geochemistry, Woods Hole Oceanographic Institution, Woods Hole, MA USA

**Keywords:** Marine chemistry, Palaeoceanography, Palaeoclimate, Physical oceanography

## Abstract

The interoceanic exchange of water masses is modulated by flow through key oceanic choke points in the Drake Passage, the Indonesian Seas, south of Africa, and south of Tasmania. Here, we use the neodymium isotope signature (ε_Nd_) of cold-water coral skeletons from intermediate depths (1460‒1689 m) to trace circulation changes south of Tasmania during the last glacial period. The key feature of our dataset is a long-term trend towards radiogenic ε_Nd_ values of ~−4.6 during the Last Glacial Maximum and Heinrich Stadial 1, which are clearly distinct from contemporaneous Southern Ocean ε_Nd_ of ~−7. When combined with previously published radiocarbon data from the same corals, our results indicate that a unique radiogenic and young water mass was present during this time. This scenario can be explained by a more vigorous Pacific overturning circulation that supported a deeper outflow of Pacific waters, including North Pacific Intermediate Water, through the Tasman Sea.

## Abstract (web summary)

Using cold-water corals, this work identifies a deep outflow of Pacific waters via the Tasman Sea during the last ice age, thus highlighting the role of this area for the interoceanic exchange of water masses on climatic time scales.

## Introduction

The exchange of water masses between ocean basins plays a key role in the volume transport and heat budgets of the global overturning circulation. The modern overturning circulation is characterized by strong southward export of deep waters from the North Atlantic into the Southern Ocean, as well as deep water formation in the high-latitude Southern Ocean^1^. Eventually, these deep waters reach the North Pacific, which hosts the oldest waters in the open ocean^2^ due to the absence of local deep convection^3^. The return of such deep waters to the surface results from diapycnal upwelling (across density surfaces) in the Pacific, which feeds upper ocean return flows to the North Atlantic Ocean^1,4^. Both diapycnal fluxes directly into the Pacific thermocline and isopycnal upwelling (along density surfaces) in the Southern Ocean contribute to that transport^1,5^. The Southern Ocean route is also important for returning old respired carbon to the surface ocean^2,5^. Together, these upwelling and return pathways balance the deep export of water from the Atlantic basin within the global overturning circulation system^1,4,5^. The return flows to the Atlantic Ocean typically follow two routes: the cold water route through the Drake Passage^6^ and the warm water route via the Indonesian Throughflow and the Agulhas Leakage south of Africa^4^.

**Fig. 1:**
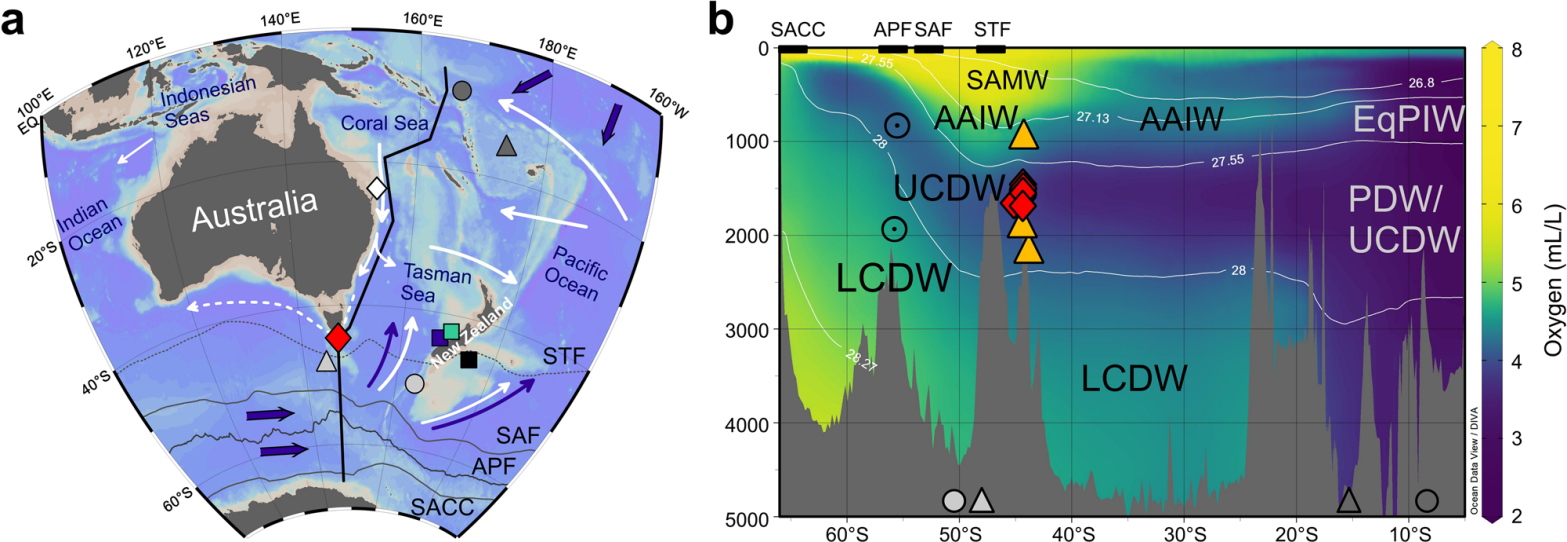
Coral sample locations and regional hydrography. **a** Map showing the coral sample locations south of Tasmania (red diamond) and locations of seawater Nd isotope profiles from stations SR3-60 (light gray triangle), TAN0803-41 (light gray circle), GeoB17019-1 (dark gray triangle), and GeoB17018-1 (dark gray circle)^38,40^. Solid white arrows indicate simplified flow paths of intermediate waters, while the dashed arrow indicates the Tasman Leakage, and dark blue arrows represent deep water flow^11,13,76,94^. The mean positions of the Subtropical Front (STF), Subantarctic Front (SAF), Antarctic Polar Front (APF), and Southern ACC front (SACC) are indicated by gray lines^14^. Colored squares indicate locations of important paleoceanographic records from sediment cores MD06-2986 (dark blue, 1477 m water depth), SO136-003GC (green, 944 m water depth)^66^, and MD97-2120 (black, 1210 m water depth)^65^. The white diamond indicates the location of core FR1/97 GC-12 (990 m water depth)^85^. **b** Oxygen concentration section^95^ (along the black line in **a**), highlighting the major subsurface water masses in the study area: Subantarctic Mode Water (SAMW), Antarctic Intermediate Water (AAIW), Upper Circumpolar Deep Water (UCDW), Lower Circumpolar Deep Water (LCDW), Pacific Deep Water (PDW), and Equatorial Pacific Intermediate Water (EqPIW) representing a mixture of AAIW originating from the Southeast Pacific and upwelled PDW^13,38^. Thin black lines indicate surfaces of neutral density anomalies γ^n^ (in kg/m^3^)^96^. Yellow triangles and red diamonds depict sampling depths of modern and fossil corals, respectively. Ocean fronts and locations of water column profiles (gray circles and triangles) as in **a**. Base map in **a** and oxygen section in **b** generated with ODV software^97^.

More recently, a third route has been recognized south of Tasmania, which is known as the Tasman Leakage and originates from the poleward transport of predominantly subsurface Pacific waters along the East Australian margin^7–12^ (Fig. 1a). The southward-flowing subsurface waters contain a local variant of Antarctic Intermediate Water (AAIW)^9,10,12^ that crossed the South Pacific Gyre after formation in the Southeast Pacific^13^. Most of the southward-flowing waters turn eastwards at the Tasman Front near ~35°S, but a proportion flows west around the Tasman margin towards the Indian Ocean^10,11^ (Fig. 1a). This outflow at intermediate depths is more saline than AAIW in the Southern Ocean^10^ and comprises both eddy transport and a non-eddy component^11^. The westward-flowing Tasman Leakage waters are bound to a narrow corridor due to bathymetric constraints and the frontal system of the Antarctic Circumpolar Current (ACC), which dictates predominantly eastward transport of water masses to the south of Tasmania^7,10,14^ (Fig. 1a). From the Indian Ocean, the Tasman Leakage waters follow the warm water route via the Agulhas Leakage^4,15,16^ and may represent up to ~50 % of the upper ocean return flow to the North Atlantic^17^. Unlike the Indonesian Throughflow and Drake Passage routes, the intermediate waters of the Tasman Leakage can preserve their hydrographic properties during transit^9,10^, thus providing a distinct pathway for the transfer of climatic signals from the Pacific to the Indian and Atlantic oceans^10^.

On daily to decadal timescales, the modern Tasman Leakage shows highly variable transport, which has been related to changes in wind forcing^7,11,12,16^. As such, the outflow can respond sensitively to short-term changes in external forcing, similar to other bottlenecks in the global overturning circulation system^7,11,15,18^. On multi-decadal to orbital timescales, changes in water flow through these various gateways have been proposed to play an important role in modulating the distribution of heat and salt between the ocean basins^19–22^. Moreover, the intensity of the shallow Pacific outflow via the Indonesian Seas has been directly linked to the overturning circulation strength in the Atlantic^23^ and to deep water inflow and upwelling rates in the Pacific basin^24^. These studies, therefore, indicate a potential mechanistic connection between the upper-ocean Pacific outflow dynamics and the deep branch of the global overturning circulation. Considering the role of Tasman Leakage waters in re-supplying the Atlantic overturning circulation today^17^, circulation changes at the Tasman margin may represent a critical component in the global overturning circulation system over glacial-interglacial timescales.

Previous research has focused on reconstructing Pleistocene changes in hydrography and transport through the Indonesian Seas^25–27^, the Agulhas Leakage^19,28,29^, and the Drake Passage^22,30–34^. The combined evidence from these studies suggests that the interoceanic exchange of water masses through the oceanic choke points was reduced during the Last Glacial Maximum (LGM: 18–24 ka BP), yet the overturning circulation was more active in the Pacific Ocean at the LGM^35^. One possibility is that changes in the outflow south of Tasmania could have compensated for reductions in the strength of the Indonesian Throughflow, as suggested for centennial to millennial timescales^12^. However, it has proven challenging to test this idea for the glacial ocean, because tracing changes in the provenance of intermediate and deep water masses is difficult with traditional paleoceanographic tools in this highly dynamic region.

**Fig. 2:**
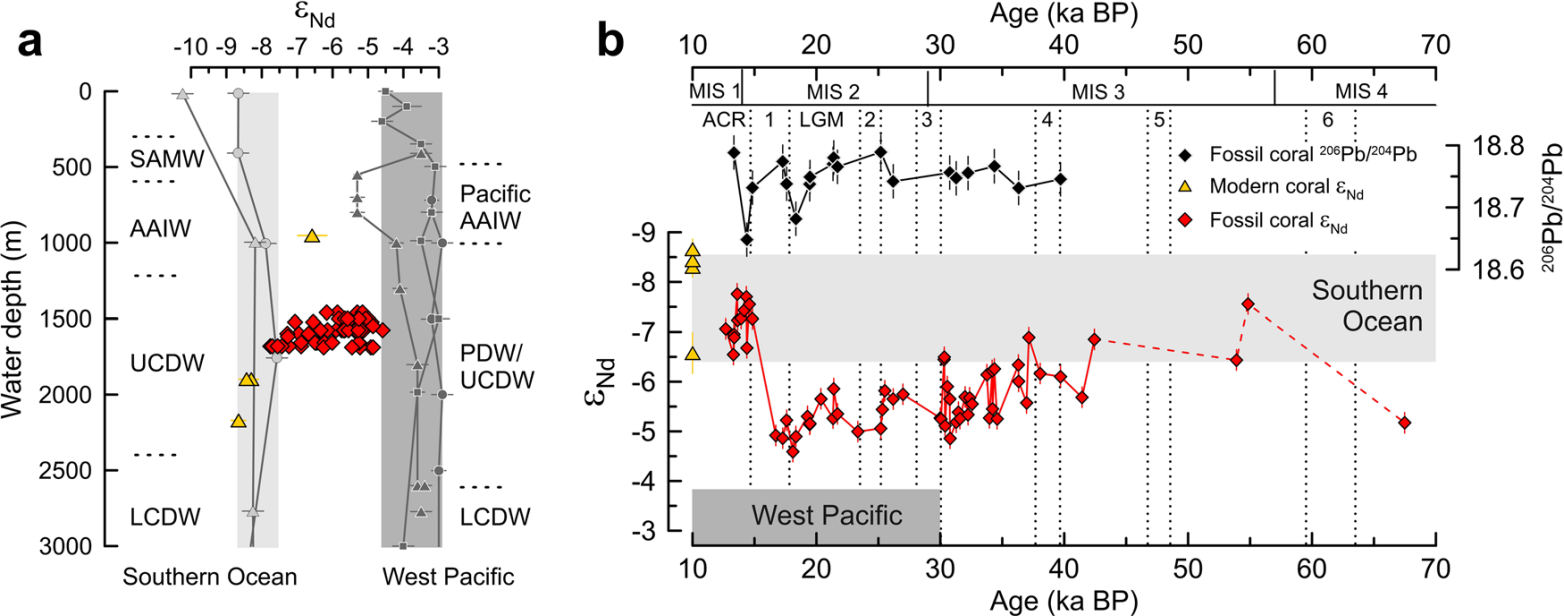
Modern and fossil Tasman cold-water coral data. **a** Coral Nd isotope data (symbols and error bars as in **b**) compared to modern seawater Nd isotope profiles from the Southern Ocean^40^ and West Pacific^38^ (symbols as in Fig. 1). Light and dark gray bars indicate the range of Nd isotope signatures (ε_Nd_) of water masses between 400 and 3000 m water depth in the Southern Ocean^40^ and in the West Pacific^38^, respectively. Also shown is a profile from North Pacific station BO-1 (gray squares; 40°N, 160°E)^98^. Error bars for seawater Nd isotope data represent the 2 s uncertainties reported in the original publications^38,40,98^. SAMW Subantarctic Mode Water, AAIW Antarctic Intermediate Water, UCDW Upper Circumpolar Deep water, LCDW Lower Circumpolar Deep Water, PDW Pacific Deep Water. **b** Time series of fossil coral ε_Nd_ compared to Pb isotope (^206^Pb/^204^Pb) data from the same specimens^50^. Error bars represent the analytical uncertainties of coral Nd (Methods) and Pb isotope data^50^. A light gray bar outlines the 2 SD of Nd isotope compositions of CDW from the Indian Ocean and the Drake Passage between 10 and 70 ka BP^34,57,59^ (see Fig. 3b for more details). The dark gray bar represents the 2 SD of past seawater Nd isotope compositions from north of the Tasman Sea for the time interval between 10 and 30 ka BP^71,72^. The numbers and boundaries of Marine Isotope Stages (MIS) are shown along the top axis. Dotted lines and numbers indicate Heinrich Stadials. ACR Antarctic Cold Reversal, LGM Last Glacial Maximum. Ages are reported in thousand years before the present (ka BP, present = 1950). Modern coral data include one replicate (see Supplementary Data 1).

Here we use the neodymium (Nd) isotope composition of the aragonitic skeletons of cold-water corals collected from intermediate water depths south of Tasmania (Fig. 1) to test the idea that circulation changes in this region played a role in the interoceanic exchange of water masses during the last glacial period between ~68 and 12.7 ka BP. At a broad scale, the modern distribution of Nd isotopes in the deep ocean is dominated by mixing between unradiogenic North Atlantic Deep Water (NADW) (ε_Nd_ ~−13)^36^ and radiogenic Pacific-derived water masses (ε_Nd_ ~−3 to −4)^37,38^. Mixing between these water masses in the ACC forms Circumpolar Deep Water (CDW), which has intermediate Nd isotope compositions (ε_Nd_ ~−8)^39,40^. The Nd isotope composition of upwelling Upper CDW (UCDW) is largely preserved during the transformation to AAIW, and is similar in composition to Lower CDW (LCDW), which results in a relatively uniform vertical distribution of Nd isotopes in the modern Southern Ocean water column^39–42^ (Fig. 2a). However, this Southern Ocean Nd isotope homogeneity did not persist back in time^33,34^, indicating the potential of using this tracer to explore past changes in ocean circulation and structure. Specifically, a glacial Nd isotope record from south of Tasmania could reveal variability in Pacific water contributions to this region, subject to constraints on past Nd cycling and Nd isotope compositions in the Pacific Ocean. In turn, such evidence would enable a unique assessment of links between the outflow of Pacific waters via the Tasman Sea and large-scale glacial-interglacial ocean circulation changes.

## Results and discussion

### Sample locations

For this study, we selected 62 fossils and three late Holocene (referred to as modern) cold-water coral specimens of *Desmophyllum dianthus* that were collected from south of Tasmania (~44°S and ~147°E) (Fig. 1a) using the remotely operated submergence vehicle JASON during cruise TN-228. The three modern corals^43^ provide a vertical transect from 951 to 2170 m water depth (Supplementary Data 1), sampling both AAIW and UCDW, which are approximately separated by the neutral density surface γ^n^ of 27.55 kg/m³ at ~1200 m water depth^13^ (Fig. 1b). The AAIW layer in the study area is occupied by different variants of AAIW, including northward-flowing Southern Ocean AAIW and southward-flowing Pacific AAIW, which is further modified along the East Australian margin by the incorporation of Tasman Sea thermocline waters to form Tasman Sea AAIW^13^ (Fig. 1). The fossil coral specimens were collected from a narrow range of 1460 to 1689 m water depth^43,44^, which in the modern ocean is occupied by UCDW and corresponds to a neutral density γ^n^ of 27.75 – 27.85 kg/m³ (refs. 7,40,.) (Fig. 1b). We recognize that the water mass structure and properties may have changed in the past, but use the nomenclature of the modern water masses to describe past oceanographic changes at the depth levels presently occupied by these water masses.

### Significance of the Tasman coral Nd isotope signal

The modern corals range from relatively radiogenic ε_Nd_ values of −6.6 ± 0.4 at 951 m water depth to less radiogenic ε_Nd_ values of −8.3 and −8.7 ± 0.2 at ~1900 and ~2170 m depth, respectively (Fig. 2a and Supplementary Data 1). The fossil corals were collected from the narrow depth range of 1460 to 1689 m and covered the later part of the last glacial period from 67.5 to 12.7 ka BP^44^, revealing ε_Nd_ variability between −4.6 ± 0.2 and −7.7 ± 0.2 (Fig. 2; Supplementary Data 1). The record is characterized by a long-term trend from ε_Nd_ values of −7.5 ± 0.2 during early Marine Isotope Stage (MIS) 3 towards maximum ε_Nd_ values of −4.6 ± 0.2 during late MIS 2. Superimposed on the long-term evolution, the corals indicate abrupt centennial to millennial-scale Nd isotope shifts, with deviations of up to ~1.5 ε_Nd_ units from the overall trend line (Fig. 2b). During the deglaciation, we observe a large shift from ε_Nd_ = −4.9 ± 0.2 at 16.7 ka BP, within early Heinrich Stadial 1 (HS 1), to ε_Nd_ = −7.3 ± 0.2 at 14.8 ka BP, around the end of HS 1. Those less radiogenic values characterize the Antarctic Cold Reversal (ACR) up to the youngest analyzed sample at 12.7 ka BP (Fig. 2b).

Rigorously cleaned skeletons of cold-water coral *D. dianthus* have been shown to reliably record ambient seawater Nd isotope signatures^45,46^. Here we add three modern corals from the Tasman continental margin to the existing calibration datasets. The Nd isotope compositions of the corals at ~1900 and ~2170 m water depth are in excellent agreement with the mean Southern Ocean seawater ε_Nd_ value of −8.2 ± 0.9 for UCDW (2 SD, *n* = 41)^40^, and with the most proximal seawater data^40^ (Fig. 2a). The modern coral from intermediate water depths has an ε_Nd_ value of −6.6 ± 0.4, which is more radiogenic than the nearby Southern Ocean AAIW ε_Nd_ values of −8.1 ± 0.3 (2 SD, *n* = 3) measured on modern seawater^40^. This difference can be attributed to the mixing between northward-flowing AAIW derived from the Southern Ocean and southward-flowing intermediate waters, which deliver more radiogenic Nd from the West Pacific region (ε_Nd_ ~−5)^38^ to the Tasman margin^10–13^ (Figs. 1, 2a). Overall, our data confirm that the skeletons of *D. dianthus* record ambient seawater Nd isotope compositions and indicate the value of Nd isotopes for tracing the presence of Pacific versus Southern Ocean water masses in the study area.

The Nd isotope composition of local seawater in the past could potentially have been modified by changes in particulate and/or solute fluxes from nearby land masses. While terrestrial input fluxes from the South Island of New Zealand into the eastern Tasman Sea were largely invariable during the last glacial-interglacial cycle^47^, changes in fluvial activity and dust emissions in Australia have been documented and represent a possible source of radiogenic Nd (ε_Nd_ > ~−5) to the study area^48,49^. To evaluate possible changes in local Nd inputs in the past, it is informative to look at lead (Pb) isotopes in the same Tasman corals^50^. Due to its higher particle reactivity, dissolved Pb has a shorter residence time than Nd in seawater^51^, such that changes in local input fluxes and sediment-seawater interaction are expected to be more readily reflected in the authigenic seawater-derived Pb isotope composition of the Tasman corals^50^. Past seawater Pb isotope reconstructions from a subset of the corals spanning ~40 to 12 ka BP show no correspondence with our Nd isotope data (*R*² = 0.03; Fig. 2b)^50^, which argues against local processes as a major control on the reconstructed Nd isotope changes. Moreover, the modern seawater and coral Nd isotope datasets do not indicate a significant influence of boundary exchange in the study area^40^. This assertion is consistent with observations from other highly dynamic regions of the Southern Ocean, including the Drake Passage where UCDW is forced through a narrow channel of rocks and sediments with relatively radiogenic Nd isotope compositions^46^. The Drake Passage experienced pronounced glacial-interglacial changes in both ocean circulation and the local input of reactive lithogenic material, with no indication that boundary exchange controlled the Nd isotope composition of past seawater^33,34^. Consequently, there is no reason to expect that boundary exchange was more pronounced in the study area during the last glacial period, so our data are best explained by a primary control from changes in water mass sourcing on a regional to basin-scale (Fig. 2a).

### Origin of the radiogenic Nd isotope signal

**Fig. 3:**
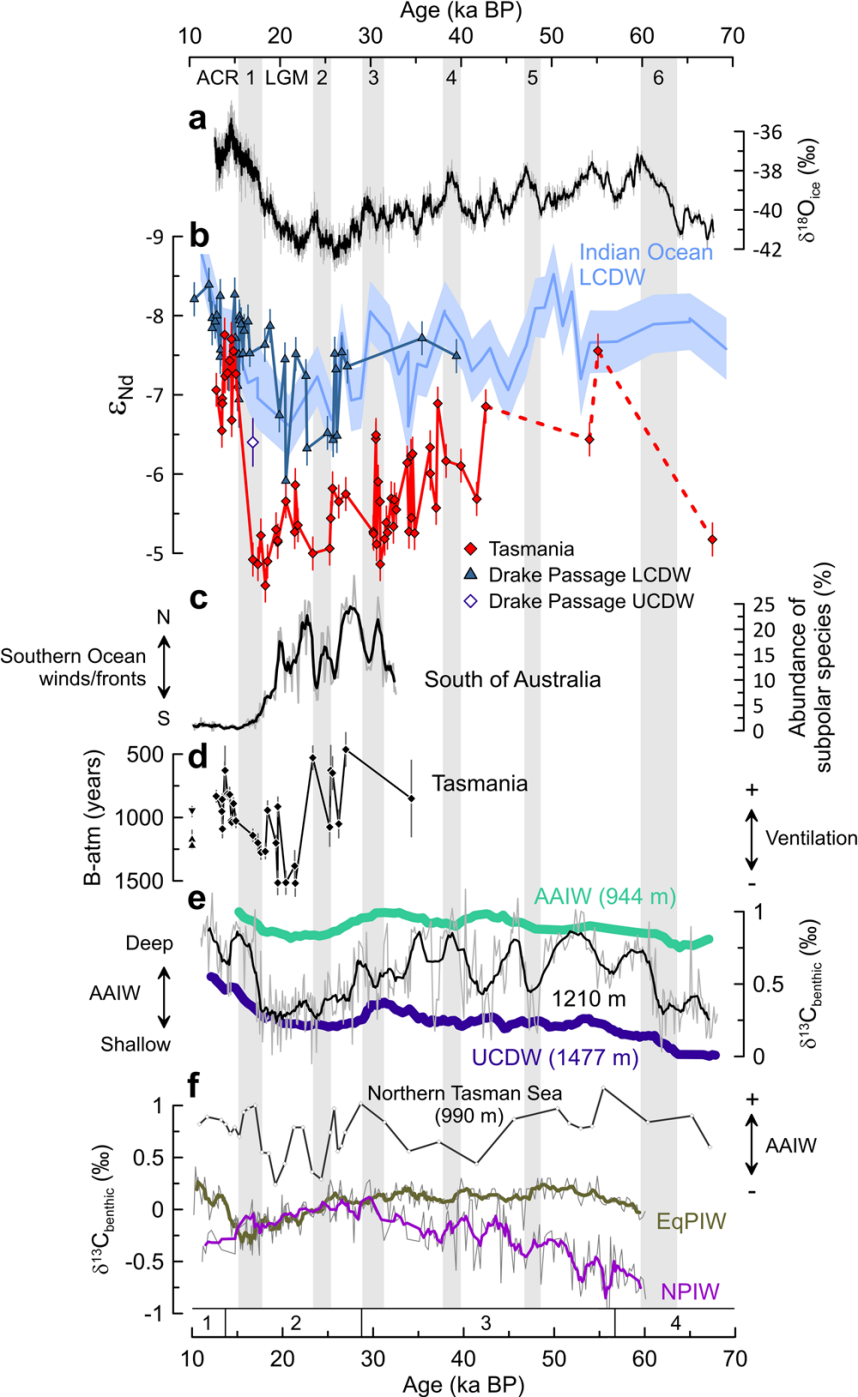
Mid-depth Nd isotope evolution south of Tasmania in paleoceanographic context. **a** WAIS Divide ice core oxygen isotope (δ^18^O) data (thin gray line) with a five-point running mean (thick black line)^99^. **b** Mid-depth cold-water coral Nd isotope (ε_Nd_) data from south of Tasmania (this study) compared to Drake passage Lower Circumpolar Deep Water (LCDW)^34^ and Upper Circumpolar Deep Water (UCDW)^30^ and Indian Ocean LCDW from core SK129-CR2 (light blue)^57,59^. Error bars and light blue shading represent the respective 2 SD uncertainties. **c** Abundance of subpolar species (light gray; five-point running mean in black) from core MD03-2611 as an indicator of changes in Southern Ocean front positions south of Australia^21^. **d** Tasmanian cold-water coral benthic-atmosphere (B-atm) radiocarbon age offsets and their propagated uncertainties (see also caption of Fig. 4) for fossil corals (black diamonds)^44^ and for the modern UCDW (black triangles) and AAIW corals (inverted black triangle)^43^ included in this study (Figs. 1b, 2a, b). **e** Benthic carbon isotope (δ^13^C) data from eastern Tasman Sea cores MD06-2986 (UCDW, 11-point running mean in dark blue) and SO136-003GC (AAIW: Antarctic intermediate water; 11-point running mean in green)^66^, compared to Southwest Pacific core MD97-2120 (light gray; 11-point running mean in black)^65^. Arrow indicates trends in AAIW depth, which is shallower than 1210 m when the MD97-2120 record converges with UCDW compositions. **f** Benthic δ^13^C data from northern Tasman Sea core FR1/97 GC-12^85^ (radiocarbon dates re-calibrated using CALIB 8.1 software^100^ and Marine20 calibration curve^101^) compared to a spliced record of North Pacific cores SO201-2-101KL and SO201-2-85KL^84,86^ (light gray; five-point running mean in purple), representing a North Pacific Intermediate Water (NPIW) source signal, and δ^13^C from sub-thermocline-dwelling foraminifera *G. hexagonus* in ODP 1240 (light gray; five-point running mean in brown), recording the composition of Equatorial Pacific Intermediate Water (EqPIW). The convergence of the latter two records has been interpreted as NPIW expansion into the Equatorial Pacific^84^. Heinrich Stadials are numbered and highlighted with gray bars along the top axis. LGM Last Glacial Maximum. ACR Antarctic Cold Reversal. The numbers and boundaries of Marine Isotope Stages 1–4 (MIS) are shown along the lower axis according to Fig. 2b.

Neodymium isotope signatures of ~−5 during the late glacial interval (25.3–16.7 ka BP) are approximately three ε_Nd_ units higher than modern seawater values at the coral sampling locations (Fig. 2b) and mark the culmination of a long-term evolution during MIS 3 and MIS 2 that corresponds to a global cooling trend (Fig. 3a, b). Building on our arguments above, we consider four possible scenarios to explain the highly radiogenic Nd isotope composition of late glacial seawater south of Tasmania: (i) changes in the composition of UCDW in the Southern Ocean, (ii) enhanced admixture of water masses from below, (iii) local deepening of a more radiogenic glacial version of AAIW, and (iv) enhanced advection of intermediate water masses from the West Pacific.

In the modern ocean, the dominant water mass at the fossil coral sampling locations is UCDW (Figs. 1b, 2a), and a northward shift of the ACC frontal system in the Indo-Pacific sector of the Southern Ocean^21,52^ (Fig. 3c) could be expected to have reinforced the presence of UCDW at the sampling locations during the late glacial interval. While Nd isotope reconstructions for glacial UCDW have been attempted using foraminifera in sediment cores east of New Zealand^53,54^, the records from this area are compromised by an offset of ~1–2 ε_Nd_ units between the measured Holocene signal^53,54^ and nearby modern seawater values^42^, which precludes a direct comparison to our coral-based record. In contrast, reconstructions from UCDW depths in the Atlantic sector of the Southern Ocean show good agreement between late Holocene foraminifera values and modern seawater, and indicate late glacial ε_Nd_ values of ~−7.5 for UCDW^55^. A more representative Southern Ocean UCDW signal is provided by Drake Passage cold-water corals, with a single specimen from UCDW depths recording an ε_Nd_ value of −6.4 during HS 1^30^. This UCDW value integrates the circumpolar flow after interaction with the radiogenic water masses of the Pacific, at a time when unradiogenic water masses from the North Atlantic exerted a reduced influence in the Southern Ocean^30^. However, a Nd isotope composition of ~−6.4 for UCDW in the Southern Ocean is not sufficiently radiogenic to explain late glacial ε_Nd_ values of ~−5 recorded at the Tasman margin (Fig. 3b).

The late glacial Nd isotope composition of LCDW was more radiogenic than in the modern ocean, which could reflect a reduced presence of unradiogenic NADW in the glacial deep ocean^34,56–60^, changes in the composition of NADW^61,62^ and/or changes in the interaction with Pacific waters^54,63^. Upwelling along sloping isopycnals provides an efficient and direct route for LCDW to reach the upper water column of the Southern Ocean^1,5^ (Fig. 1b). Therefore, a northward shift of the ACC frontal system^21,52^ (Fig. 3c) could potentially have increased the exposure of the Tasman corals to LCDW during the late glacial interval (Fig. 1b). However, glacial LCDW was characterized by relatively unradiogenic ε_Nd_ values of ~−6 to −8, as evidenced by reconstructions from the South Pacific^60^, the Drake Passage^34^, and the deep Indian Ocean^57,59^ (Fig. 3b). These values are too unradiogenic to drive concurrent ε_Nd_ values south of Tasmania to ~−5. Importantly, an increased presence of LCDW at the coral sampling locations would require a northward shift of the ACC frontal system and the associated isopycnals by more than 10° latitude, which would push both the Subantarctic Front and the Antarctic Polar Front against the Tasman margin (Fig. 1b). Such a scenario is difficult to reconcile with the modern observation that the South Tasman Rise and Campbell Plateau force the deep-reaching fronts of the ACC into a circumpolar trajectory to the south of the coral sampling locations^14^ (Fig. 1a). Moreover, LCDW was characterized by a low radiocarbon content at the LGM, with a B-atm offset (i.e. the offset between marine benthic and atmospheric ^14^C ages^64^) of ~3000 years, whereas concurrent B-atm values at the Tasman margin fluctuated around ~1200 years^44^ (Fig. 3d). Thus, we exclude the exposure to LCDW as a primary control on the Nd isotope evolution recorded by the cold-water corals.

**Fig. 4:**
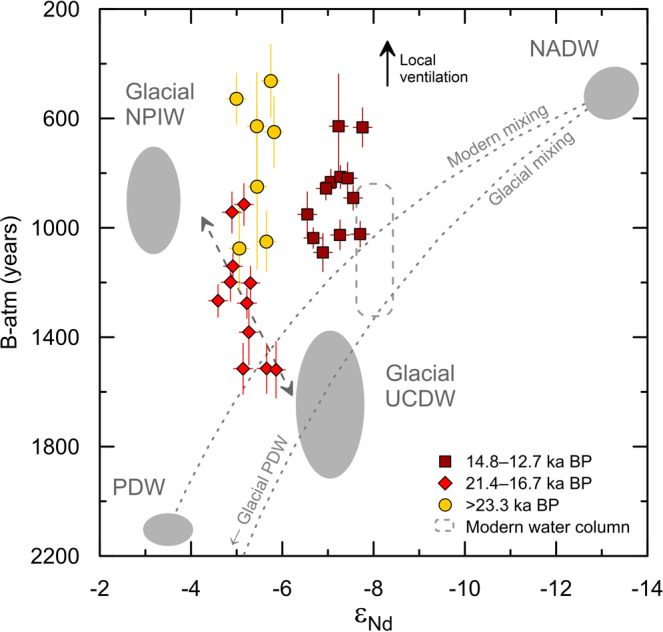
Tasman cold-water coral data in radiocarbon–Nd isotope (ε_Nd_) space. The benthic-atmosphere radiocarbon (^14^C) age offsets (B-atm) were calculated using an atmospheric ^14^C age of 0 years (1950 AD) for the modern seawater values^2,102^. For past B-atm, we used coral ^14^C ages^44^ and IntCal20 atmospheric ^14^C ages^102^. Atmospheric ^14^C values were calculated as averages of the reported 1 s coral calendar age uncertainty range^44^. Error bars represent 1 s propagated uncertainties for B-atm age offsets and 2 SD uncertainties for ε_Nd_. Modern water column data comprise regional Antarctic Intermediate Water (AAIW), Upper Circumpolar Deep Water (UCDW), and Lower Circumpolar Deep Water (LCDW) properties^2,40^. Hypothetical mixing calculations (dotted lines) use the following endmembers: modern North Atlantic Deep Water (NADW, ε_Nd_ = −13.2, [Nd] = 17.6 pmol/kg, B-atm = 500 years, DIC [dissolved inorganic carbon] = 2160 μmol/kg)^2,36^; modern Pacific Deep Water (PDW, ε_Nd_ = −3.5, [Nd] = 44.4 pmol/kg, B-atm = 2100 years, DIC = 2350 μmol/kg)^2,37^. Note that these hypothetical mixing lines do not account for non-conservative processes along the water mass flow paths. Glacial PDW was defined using an LGM B-atm age offset of 3000 years^64^ and otherwise modern PDW parameters due to the similarity of glacial and modern PDW Nd isotope compositions^54,71,72^. Glacial UCDW was estimated based on the Drake Passage coral Nd isotope^30,34^ and radiocarbon data^31,69,103^. Glacial North Pacific Intermediate Water (NPIW) properties at its source are estimated based on modern Nd isotope compositions^37,38^ and LGM radiocarbon ages^35^. A dashed gray arrow indicates mixing between glacial UCDW and glacial NPIW. Local ventilation (e.g., via deepening of Southern Ocean AAIW) would lead to lower B-atm and relatively invariant ε_Nd_ values (black arrow). Where paired radiocarbon–Nd isotope data were not available from the same coral specimen, we matched the Nd isotope data with radiocarbon data from nearby corals of the same age (i.e., within age uncertainty and with <100 m depth difference).

If glacial AAIW in the Southern Ocean had a significantly more radiogenic Nd isotope composition than today, local deepening of the AAIW layer could provide a possible explanation for the radiogenic Nd isotope signatures at the coral sampling locations (Fig. 1b). A deepening of Southern Ocean AAIW to reach the coral sampling depths would be consistent with the relatively well-ventilated signal reconstructed from radiocarbon^44^ (Fig. 3d). However, AAIW is formed from UCDW, which was significantly less radiogenic (ε_Nd_ ~−7)^30,55^ than our coral data (ε_Nd_ ~−5) during the late glacial interval (Fig. 4). Recent work from the Drake Passage shows that the Nd isotope signal of UCDW is generally preserved during water mass transformation in the Southern Ocean upper overturning cell^33^. Moreover, based on modern seawater data it can be expected that any local influences would drive the surface waters between Australia and Antarctica to even less radiogenic values than the upwelling UCDW ε_Nd_ signal^40^. Therefore, a deep variant of AAIW forming in the Southern Ocean south of Australia would be unable to explain the shift to such radiogenic Nd isotope signatures at the coral sampling locations during the last glacial period. In addition, rather than a glacial deepening, independent evidence from stable oxygen and carbon isotopes and from redox-sensitive proxies (rhenium and uranium) suggests a glacial shoaling of Southern Ocean AAIW in the study area^65–67^ (Fig. 3e).

A more radiogenic variant of AAIW is present in the modern West Pacific^38^ (Fig. 2a). This variant is mainly supplied by AAIW formation in the Southeast Pacific and modified to Tasman AAIW upon entering the Tasman Sea from the north^13,67^ (Fig. 1a). A recent study proposed an increased presence of Tasman AAIW over the Challenger Plateau in the eastern Tasman Sea (west of New Zealand) during the LGM^67^. Nevertheless, previous work from the main AAIW formation region in the Southeast Pacific^68,69^, as well as in areas influenced by Tasman AAIW (i.e., eastern Tasman Sea and Bay of Plenty)^66,67,70^ (Fig. 1a), are inconsistent with a deepening of Tasman AAIW to reach water depths of ~1500–1700 m during the LGM.

Consequently, the highly radiogenic Nd isotope signatures observed in late glacial corals from south of Tasmania were probably not the result of changes in the composition and/or depth ranges occupied by LCDW, UCDW, or AAIW. Instead, we consider the advection of radiogenic water masses from the Pacific Ocean north of the Tasman Sea to provide the most likely explanation (Fig. 1b). Importantly, this scenario requires a shift in the glacial circulation compared to the modern-day.

### North Pacific water mass signals at the Tasman margin during the last glacial period

The circulation between the Tasman Sea and the West Pacific is restricted by complex bathymetric features (Fig. 1a), which confine the exchange of waters below ~1500 m water depth to narrow deep channels. The density range of the waters bathing our fossil coral sampling locations is currently occupied by UCDW and PDW, which share similar ε_Nd_ values of ~−3 to −4 in the modern West Pacific^38^ (Figs. 1, 2a). Reconstructed glacial ε_Nd_ values of ~−2.5 to −4 for mid-depth waters (~2–2.6 km water depth) in the West Pacific from north of the Tasman Sea^71,72^ are indistinguishable from those modern values (Fig. 2b). However, while the radiogenic Nd isotope compositions of the Tasman corals approached PDW values during the late glacial interval (ε_Nd_ = −2 to −4)^54,71^, they were associated with much younger ventilation ages (Fig. 4). At the LGM, PDW was characterized by a pronounced radiocarbon depletion (B-atm ~3000 years)^64^, while the coral data from the Tasman margin show B-atm values of ~1200 years^44^ (Fig. 3d). Therefore, an increased presence of PDW is unsuitable to explain the trend towards more radiogenic Nd isotope compositions at the Tasman margin during the last glacial period (Fig. 4). The combined radiocarbon and Nd isotope evidence suggest that our LGM and early deglacial coral data lie on a mixing trend between an unradiogenic and poorly-ventilated source represented by glacial Southern Ocean UCDW and relatively well-ventilated and radiogenic Pacific-derived waters (Fig. 4). We hypothesize that this Pacific-derived outflow signal was driven by an expansion of North Pacific Intermediate Water (NPIW) during the last glacial period.

**Fig. 5:**
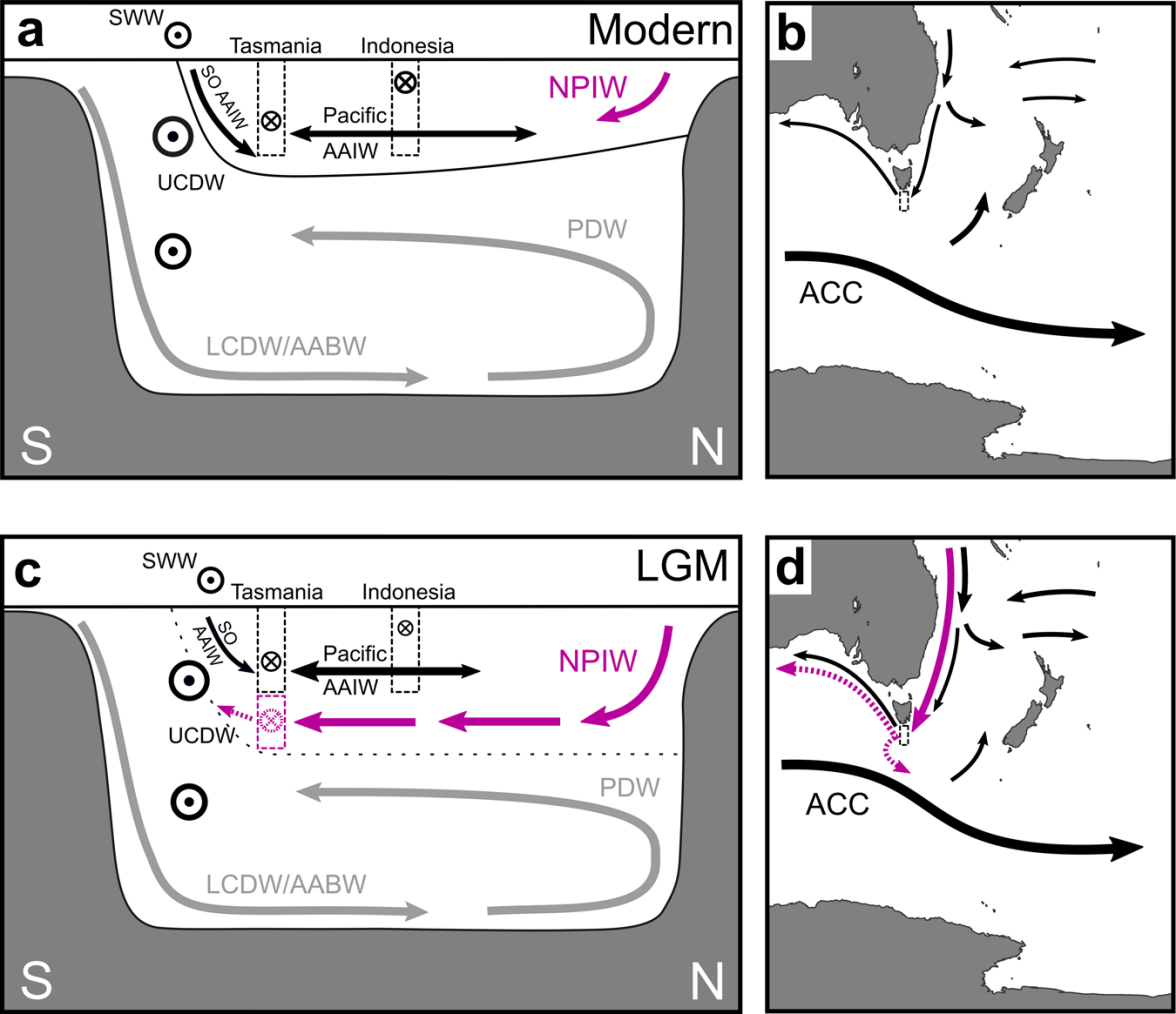
Schematic of inferred circulation changes between the modern-day and the Last Glacial Maximum (LGM). Cross-sections show the Pacific Ocean circulation for **a** the modern-day and **c** the LGM. Maps focus on the intermediate-depth circulation in the study area near Tasmania for **b** the present-day (see also Fig. 1a) and **d** the LGM. Base maps in **b**, **d** produced with ODV software^97^. Water mass pathways are indicated by arrows: Antarctic Intermediate Water (AAIW, black) and North Pacific Intermediate Water (NPIW, purple). SO: Southern Ocean. Note that the eastward flow of the Antarctic Circumpolar Current (ACC) (circled dots in **a** and **c**) comprises also Upper Circumpolar Deep Water (UCDW) and Lower Circumpolar Deep Water (LCDW) (see also Fig. 1b for more details). Note also that the two possible pathways of glacial Tasman outflow waters are indicated by stippled lines in **c**, **d** and that the purple arrows in **c**, **d** do not represent pure NPIW, since it is influenced by the admixture of other ambient water masses during its southwards transport (see main text). Gray arrows in **a**, **c** indicate the deep overturning, where upwelling of LCDW and Antarctic Bottom Water (AABW) feeds into the southward return flow of Pacific Deep Water (PDW). Flow strengths are schematically represented by the thickness of arrows (meridional flows) or the size of the circled dots/crosses (zonal flows). The solid black line in **a** represents the intermediate water layer ventilated primarily by AAIW and NPIW in the modern Pacific Ocean, while the stippled black line in **c** indicates the deepened LGM analogue. Dashed boxes indicate the locations and approximate depth extents of Pacific outflow along the Tasman margin and through the Indonesian Seas. The position of the Southern Westerly Winds (SWW) is also shown with circled dots.

In the modern ocean, NPIW is characterized by ε_Nd_ values of ~−3^37,38^, but is confined to the North Pacific^73,74^ (Fig. 5a) as it leaves the Pacific through the Indonesian Seas^74^ above the sill depth of ~1300 m^18,75,76^. Its incorporation into the deep Tasman outflow during the last glacial period would thus require a mechanism that enables the cross-equatorial flow of NPIW (Fig. 5c). Modeling studies indicate that the formation of intermediate water masses in the North Pacific and/or their expansion across the Pacific basin may be sensitive to the strength of the Indonesian Throughflow^77,78^. Glacio-eustatic sea-level fall, the associated shelf exposure in the Indonesian Seas, and changes in the coupled ocean-atmosphere system of the tropical Indo-Pacific probably led to a reduction in the Indonesian Throughflow during glacial periods^25,27^ (Fig. 5c). An additional reduction of the Indonesian Throughflow may have arisen from a possible link with the weakened overturning circulation in the Atlantic Ocean under glacial boundary conditions^23^.

Consistent with those modelling predictions^77,78^, proxy evidence points to the existence of a chemical divide at about 2000 m water depth in the Pacific Ocean at the LGM, with the upper part of the water column dominated by relatively well-ventilated waters^35,79,80^. This ventilated state has been attributed to a deepening and intensification of NPIW formation^35,79–81^, which was presumably related to subpolar gyre dynamics and reduced freshwater fluxes to the North Pacific^35,82^. The expanded glacial NPIW signal was detected in reconstructions from the equatorial Pacific^83,84^ (Fig. 3f) and has been proposed to have spread towards the Southern Ocean predominantly along the western boundary of the Pacific^35^. A record of benthic carbon isotopes from 990 m water depth on the east Australian margin (northern Tasman Sea) (Fig. 1a) reveals values that were originally interpreted to result from changes in mixing between AAIW and UCDW^85^. However, glacial NPIW and glacial UCDW are characterized by rather similar δ^13^C^66,84^ (Fig. 3e, f). Thus, the northern Tasman Sea record may also be explained by an increased influence of southward-flowing glacial NPIW (low δ^13^C)^84,86^ alternating with shallower AAIW (higher δ^13^C)^66,70,85^ at these depths during the last glacial period (Fig. 3f). Notably, the peak in NPIW formation during HS 1^20,86,87^ coincides with the most radiogenic Nd isotope compositions and improved ventilation recorded south of Tasmania (Fig. 3b, d). Hence, we propose that the long-term glacial trend towards more radiogenic Nd isotope compositions south of Tasmania was linked to the expansion of glacial NPIW and its incorporation into an outflow through the Tasman Sea below the depth range of modern Tasman Leakage waters. If this interpretation is correct, our data provide evidence that glacial NPIW reached the extratropical South Pacific.

### Glacial reorganization of Pacific Ocean circulation and inter-ocean exchange

It has previously been speculated that a reduction in the Indonesian Throughflow could be compensated, on centennial to millennial timescales, by enhanced export of Pacific water masses via the Tasman Sea^12^. Our coral data corroborate this idea and provide evidence that a glacial reorganization of ocean circulation in the Pacific could have involved the export of NPIW via the Tasman Sea (Fig. 5c, d). Such export would have been particularly efficient during times when NPIW deepened below the Indonesian Throughflow sill depth of ~1300 m^75^ (Fig. 5c). A reduced inflow of Southern Ocean AAIW into the intermediate depths of the Pacific Ocean (Fig. 5c, d), linked to a shoaling^66^ and/or weakening^65^ of Southern Ocean AAIW formation during the LGM, could have further supported the southwards expansion of NPIW within the Pacific basin^77^. We emphasize that the glacial outflow described here was found at depths significantly below the modern Tasman Leakage waters (Fig. 5a, c). Taken in combination with the possible lower boundary of Tasman AAIW near ~1000 m water depth^67,85^, our coral dataset suggests that the deep outflow extended from ~1000 to at least ~1700 m water depth during the last glacial interval. At these depths, the southwards expanding glacial NPIW was probably subject to an admixture of other water masses in the West Pacific, potentially including glacial PDW, UCDW, and different variants of AAIW (Figs. 1b, 5c, d). It is difficult to diagnose their individual contributions to the deep outflow signal at the Tasman margin from our dataset because these water masses have similar Nd isotope compositions in the modern Pacific Ocean, reflecting the non-conservative effects of local Nd inputs on the Nd isotope composition of seawater^38^. However, relatively invariant glacial-interglacial ε_Nd_ values of ~−2.5 to −4 reconstructed from sites north of the Tasman Sea^54,71,72^ (Fig. 2b) suggest that any changes in advection, boundary exchange, benthic flux, weathering inputs, or open ocean particle-seawater interactions appear to have had a minimal effect on mid-depth West Pacific Nd isotope signatures across this interval^38,47,63,71,72^. Accordingly, a glacial intermediate water mass entering the Tasman Sea from the north would probably have been characterized by ε_Nd_ values near or above ~−4, making it suitable to drive the trend towards more radiogenic glacial ε_Nd_ values at the Tasman margin.

The key finding from our work is that these radiogenic Pacific-derived water masses contributed substantially to an outflow from the Tasman Sea at water depths of ~1500–1700 m during the last glacial period. This scenario is different from the modern situation, in which the outflow is concentrated in the Tasman Leakage at depths shallower than ~1200 m^10,12,16^. We ascribe this deep Tasman outflow to a glacial mode of more active NPIW formation^35,81,84^ (Fig. 5c, d), which has been proposed to have promoted the carbon storage in the deep ocean thus contributing to the glacial reduction of atmospheric CO_2_^35^.

The deep Tasman outflow waters could have taken two possible routes after passing the coral sampling locations, with distinct implications for the glacial overturning circulation system. If the outflow waters followed the trajectory of the modern shallower Tasman Leakage waters into the Indian and Atlantic Oceans^17^ (Fig. 5a–d), they would have helped to maintain the interoceanic exchange of water masses and provided an oceanic pathway for the westward export of Pacific climate signals^10,16^. Conversely, parts of the deep Tasman outflow waters may have joined the eastward flow of the ACC (Fig. 5c, b, d), thus propagating climate signals into the Southern Ocean and influencing the nutrient availability in the Southern Ocean upon upwelling to the surface^35^. Considering the enhanced northwards deflection of circumpolar water masses in the Southeast Pacific during the late glacial interval^22,32^, such eastward-flowing waters would have contributed to a more isolated upper overturning cell in the Pacific Ocean^78,80^ rather than enhancing interoceanic exchange at that time.

While our coral data are unable to provide constraints on the relative importance of these two possible routes downstream of Tasmania, the most radiogenic ε_Nd_ values in our record suggest that the outflow of Pacific waters was most pronounced during HS 1 (Fig. 3b) when changes in atmospheric circulation and salinity feedbacks promoted convective activity in the North Pacific^20,78,87^, and when a poleward shift of Southern Ocean winds and fronts^21,52^ (Fig. 3c) may have effectively widened the westward passageway for outflow waters at the Tasman margin^7^. Accordingly, such frontal shifts may also have been responsible for the rapid Nd isotope changes on centennial to millennial timescales during MIS 2. Albeit the most radiogenic Nd isotope compositions recorded by the Tasman corals coincided with HS 1, radiogenic Nd isotope excursions could also be a sign of enhanced Pacific outflow during previous Heinrich stadials (Fig. 3b). Furthermore, our coral data clearly indicate that the structure of the Pacific Ocean was substantially reorganized at the end of HS 1. At that time, convection in the North Pacific decreased^20,86^ and the expansion of NADW supported more modern-like water column characteristics in the Southern Ocean^34,56,60^, leading to Nd isotope compositions approaching modern values south of Tasmania (Fig. 3b).

Overall, our study provides evidence that a Pacific outflow through the Tasman Sea played a dynamic role in the interoceanic exchange of water masses within the global overturning circulation system through glacial-interglacial cycles. We demonstrate a close link between changes in the deep Tasman outflow, NPIW formation, and circulation changes in the Pacific Ocean during the last glacial period. Implementing these findings into refined ocean models will improve our understanding of how the outflow of Pacific waters via the Tasman Sea affects the interoceanic exchange of climate signals, the global overturning circulation strength, and the oceanic carbon storage, over a range of timescales in the past and the future.

## Methods

### Sample preparation and analytical procedures

Neodymium isotope analyses were carried out in the MAGIC laboratories at Imperial College London following established protocols^46,88^. In brief, physically and chemically cleaned cold-water coral samples were subjected to iron co-precipitation to concentrate trace metals before separating uranium (U) and thorium (Th) for U-series dating^44^. The Nd fractions of the fossil corals were collected during U-Th separation, whereas the Nd fractions of the three modern corals were collected from the wash fraction during Pb separation^50^. All Nd fractions were purified using a two-step ion-exchange chromatography protocol for analysis by thermal ionization mass spectrometry (TIMS) as NdO^+^^88^.

Long-term TIMS results of 5 and 15 ng loads of JNdi-1 yielded ^143^Nd/^144^Nd = 0.512105 ± 0.000009 (2 SD, *n* = 110). For each analytical session, the instrumental offset was corrected by normalizing the mass-bias corrected ^143^Nd/^144^Nd ratios of samples based on the JNdi-1 reference ratio of ^143^Nd/^144^Nd = 0.512115 ± 0.000007^46,89^. The mass-bias corrected ^143^Nd/^144^Nd ratios from one analytical session were subjected to a secondary correction using ^142^Nd/^144^Nd^90^ (see also Supplementary Data 1), yielding ^143^Nd/^144^Nd = 0.512117 ± 0.000012 (2 SD, *n* = 5) for JNdi-1. The long-term external reproducibility was monitored with repeat analyses of USGS reference material BCR-2 and our in-house coral reference material during all analytical sessions, yielding ^143^Nd/^144^Nd of 0.512637 ± 0.000010 (2 SD, *n* = 34) and 0.512336 ± 0.000011 (2 SD, *n* = 25), respectively. These results are in excellent agreement with published results for BCR-2 (0.512637 ± 0.000012)^91^ and our coral reference material (0.512338 ± 0.000008)^92^. We report the 2 SD of the coral reference material as the analytical uncertainty for samples unless the internal 2SE is larger in which case the propagated error is reported. Full procedural blanks for combined U-Th/Pb and Nd separation from the aragonitic matrix ranged from 5 to 31 pg Nd, with a mean of 20 pg (*n* = 12). All Nd isotope results are reported as ε_Nd_ = ((^143^Nd/^144^Nd_sample_)/(^143^Nd/^144^Nd_CHUR_) – 1) × 10,000, where CHUR is the chondritic uniform reservoir^93^.

## Supplementary information

Description of Additional Supplementary Files

Supplementary Data 1

## Data Availability

The data generated in this study are provided in the Source Data file and available in the figshare database under 10.6084/m9.figshare.19355633.
