# Supplementary material for: A deep Tasman outflow of Pacific waters during the last glacial period
Source: Nat Commun. 2022 Jun 30;13:3763. doi: 10.1038/s41467-022-31116-7 (PMC9246942; doi:10.1038/s41467-022-31116-7)
Supplement: Supplementary file 1 — Description of Additional Supplementary Files [file 41467_2022_31116_MOESM1_ESM.pdf]

Description of Additional Supplementary Files

File Name: Supplementary Data 1
Description: Tasmanian cold-water coral data.
